# Self-Assembly of Soft and Conformable Broadband Absorbing Nanocellulose-Gold Nanoparticle Composites

**DOI:** 10.1021/acsami.4c10244

**Published:** 2024-09-23

**Authors:** Olof Eskilson, Elisa Zattarin, Jennifer Silander, Tomas Hallberg, Christina Åkerlind, Robert Selegård, Kenneth Järrendahl, Daniel Aili

**Affiliations:** † Laboratory of Molecular Materials, Division of Biophysics and Bioengineering, Department of Physics, Chemistry, and Biology, Linköping University, 581 83 Linköping, Sweden; ‡ Department of Electromagnetic Signatures, FOI-Swedish Defence Research Agency, 583 30 Linköping, Sweden; § Thin Film Physics Division, Department of Physics, Chemistry, and Biology, Linköping University, 581 83 Linköping, Sweden

**Keywords:** plasmonic materials, nanocomposite, gold nanoparticles, nanocellulose, absorptance

## Abstract

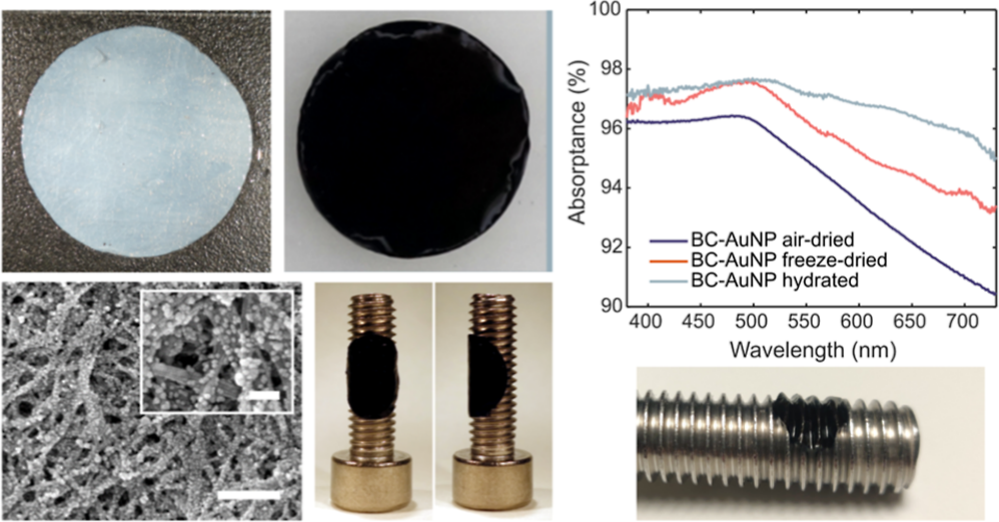

Broadband light-absorbing materials are of large interest for numerous applications ranging from solar harvesting and photocatalysis to low reflection coatings. Fabrication of these materials is often complex and typically utilizes coating techniques optimized for flat and hard materials. Here, we show a self-assembly based strategy for generating robust but mechanically flexible broadband light-absorbing soft materials that can conform to curved surfaces and surface irregularities. The materials were fabricated by adsorbing large quantities of gold nanoparticles (AuNPs) on the nanofibrils of hydrated bacterial cellulose (BC) membranes by tailoring the interaction potential between the cellulose nanofibrils and the AuNPs. The highly efficient self-assembly process resulted in very dense multilayers of AuNPs on the nanofibrils, causing extensive broadening of the localized surface plasmon resonance band and a striking black appearance of the BC membranes. The nanocomposite materials showed an absorptance >96% in both the visible and the near-infrared wavelength range. The AuNP-functionalized BC membranes demonstrated excellent conformability to curved and structured surfaces and could adopt the shape of highly irregular surface structures without any obvious changes in their optical properties. The proposed self-assembly based strategy enables the fabrication of soft and conformable broadband light-absorbing nanocomposites with unique optical and mechanical properties using sustainable cellulose-based materials.

## Introduction

Broadband light-absorbing materials with low reflectance, often referred to as ultrablack materials, are of large interest for a number of applications, including solar harvesting,^1−4^ photocatalysis,^5,6^ and low-reflection coatings.^7,8^ In nature, broadband light-absorbing materials are seen in a number of species of both plants and animals.^9−11^ In the animal kingdom, snakes,^11^ butterflies,^12^ and some birds are among the animals that can produce broadband light-absorbing features. Due to structural components, some bird-of-paradise species feathers absorbs up to 99.95% of incident light.^13^ Synthetic broadband absorbing materials can be obtained using different material combinations and fabrication techniques with the ambition of optimizing light–matter interactions to suppress transmittance and reflection while absorbing radiation at a wide range of wavelengths (λ) in a relevant range and at different incident angles.^14^ Suppression of reflection can also be accomplished by creating gradients in the refractive index at the interface of the material.^15^ Iterative reflections and scattering events inside the material combined with high absorbing structures and materials, such as plasmonic nanostructures or nanoparticles (NPs), further increase the probability of photons being absorbed.^2,3,5,6,16−18^ For example, Ng et al. deployed Au sputtering techniques to create a structured surface of Au nanotubes with gaps in the size of 85 nm and walls with a thickness of 15 nm,^6^ to accomplish this. The importance of surface structure on broadband absorptance has also previously been demonstrated by McCoy et al.,^13^ Davis et al.,^12^ Wetzel et al.,^19^ and others.^7,16,17,20^ Recent progress in advanced microfabrication techniques has facilitated development of strategies for generating broadband absorbing coatings for metals, metal oxides, and silicon oxide.^21−24^ However, the difficulties in controlling both structure and material properties over multiple length scales make the production of broadband absorptance materials very challenging and costly. In addition to the complicated processing techniques resulting in expensive materials, these strategies are primarily applicable to planar 2D surfaces and a limited number of different substrate materials. Thus, creating soft broadband absorbing materials, mimicking biological materials, remains very challenging.

**Figure 1:**
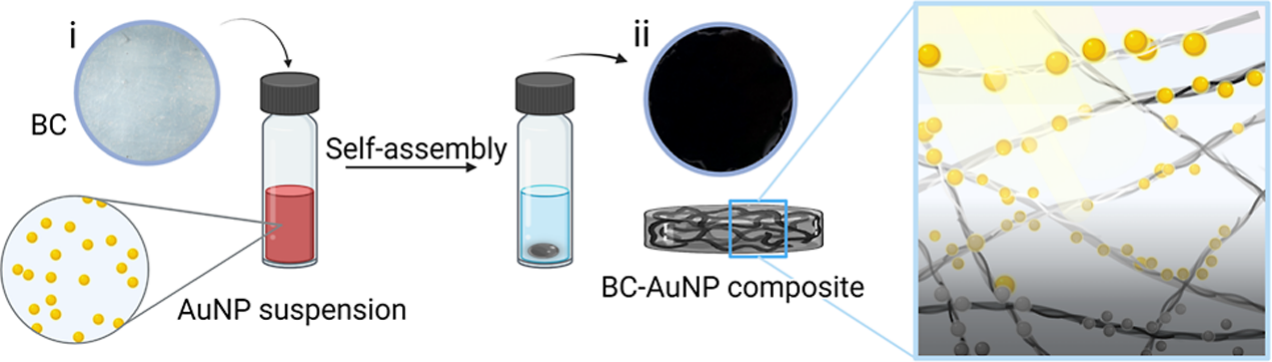
Schematic description of the self-assembly process for generating high densities of adsorbed AuNPs to bacterial nanocellulose membranes and corresponding photographs of (i) native BC and (ii) hydrated BC-AuNP membranes, ø 2 cm.

Here, we show that broadband absorbing materials can be obtained by the self-assembly of plasmonic gold nanoparticles (AuNPs) in thin bacterial cellulose (BC) membranes (Figure 1). BC is a hydrated, nanoporous, soft, and very flexible nanofibrillar cellulose material produced by certain species of bacteria such as *Komagataeibacter xylinus*.

BC fibrils have a typical diameter of 50–200 nm, resulting in a large effective surface area,^25,26^ which makes it an attractive scaffold for fabrication of nanocomposite materials. Multiple strategies have been developed for in situ synthesis of NPs, including AuNPs, in BC for generating composites with novel properties.^27−30^ However, because of the presence of BC during the in situ synthesis process, it is very challenging to control the size, geometry, and concentration of the NPs. We have recently demonstrated that it is possible to tailor the interaction potential between BC nanofibrils and inorganic NPs, including AuNPs, for self-assembly of nanocomposites with well-defined optical properties.^26^ Nonsterically stabilized NPs bind strongly to BC fibrils above a critical ionic strength of the suspensions. Because of the high-surface area of BC, large quantities of AuNPs can be adsorbed in the membranes, resulting in materials with an opaque and very black appearance.^26^ By further optimizing the assembly process and post-treatment of these nanocomposites, it is possible to obtain flexible materials with broadband light-absorption properties comparable to more complex materials obtained by using significantly more elaborate and costly fabrication techniques. The ease and scalability of manufacturing and the low cost of the components combined with the unique optical and mechanical properties of the resulting materials make them highly interesting for a wide range of applications.

Broadband absorbing coatings have been widely applied to modify stiff metal and silicon-based materials,^8,16,17,20,31,32^ providing dynamic and mechanically flexible materials with broadband absorbing properties is significantly more challenging. Broadband absorbing materials obtained by functionalization of flexible substrates such as scotch tape,^2^ or PDMS,^6^ have been demonstrated, but these strategies result in inhomogeneous materials with a risk of delamination at the joining interface. The BC-AuNP composites described herein are robust and morphologically homogeneous and display high mechanical conformability and flexibility, providing novel material characteristics and possibilities to extend the range of applications for broadband absorbing materials.

## Experimental Section

### General

All chemicals were acquired from Merck KGaA (Darmstadt, Germany) and used as procured without further purification. BC produced by *K. xylinus* was obtained from S2medical AB (Linköping, Sweden).

### AuNP Synthesis

AuNPs were synthesized using the Turkevich-method,^33^ reducing HAuCl_4_ using citrate. Glassware was thoroughly cleaned using a 1:1:5 mixture of 30% H_2_O_2_, 25% NH_3_, and ultrapure Milli-Q (MQ) water (18.2 MΩ cm^–1^), heated to 85 °C for 5 min after which the glassware was carefully rinsed in MQ water. A 200 mL solution of HAuCl_4_ was heated to a rolling boil, and the reduction was started by quickly adding 20 mL of 38.8 mM sodium citrate solution while stirring vigorously. The mixture was refluxed for approximately 15 min after which it was taken off the heat and left to cool to RT.

### Self-Assembly of AuNPs in BC

Pieces of BC were cut using a biopsy punch of 2 cm diameter and rinsed in MQ. One piece of BC (ø 2 cm) was added to a 10 mL Erlenmeyer flask with 2 mL of concentrated 195 nM AuNP suspension (ø 12 ± 1 nm). The concentrated AuNP suspensions were produced by centrifuging 2 mL of 13 nM suspension in Eppendorf tube at 12,500 rpm for 15 min after which the supernatant was discarded and pellet resuspended in citrate buffer 10 mM pH 6. The Erlenmeyer flasks containing BC and AuNP suspension were placed on an orbital shaker until AuNP suspensions were colorless, roughly 3 days. For Figure S1, one 6 mm diameter BC piece was cut using a biopsy punch and immersed in an Eppendorf tube containing a mixture of 500 μL of AuNP suspension 13 nM and 500 μL of citrate buffer 10 mM pH 6 and left to incubate on an orbital shaker for 5 days after which it was carefully rinsed in MQ to remove any unbound AuNPs.

### Drying of BC-AuNP Composites

Two drying methods were used to achieve dry BC-AuNP composites. Freeze–drying was carried out by flash freezing the BC-AuNP composites using liquid nitrogen, followed by lyophilization. For air-drying, BC-AuNP composites were kept in a desiccator cabinet for ≥12 h.

### UV–Vis–NIR Measurements

UV–vis–NIR spectra were acquired in the form of total directional hemispherical reflectance (DHR) and total directional hemispherical transmittance (DHT) using a Cary 5000 Agilent spectrophotometer (Santa Clara, CA, US), with a 150 mm diameter integrating sphere attached (Labsphere DRA-2500), the inside coated with Spectralon. The instrument was calibrated against a Spectralon diffuse reflectance standard with 99% reflectance. The sample was fixed between two black painted apertures, slightly smaller than the size of the sample. The sample beam size was reduced to fit the sample. Then, the sample was positioned vertically at the back reflectance position in the integrating sphere for the reflectance measurement and at the entrance of the integrating sphere for the transmittance measurement (Figure S6, Supporting Information). Data shown in Figure S1 was obtained using a microplate reader (Tecan Infinite M1000 Pro, Tecan Austria GmbH, Grödig/Salzburg, Austria).

### Water Absorption Capacity

8 mm BC and BC-AuNP composites were dried by air drying or freeze–drying (*n* = 3). The sample dry weight was recorded (*W*_d_) prior to submerging the samples in distilled water at room temperature, where the membranes were allowed to swell for predetermined time intervals. Excess water was then removed by pat drying with a wet tissue, and the instantaneous weight was recorded (*W*_i_). Water absorption was calculated as follows:
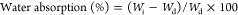


Statistical analysis was carried out using GraphPad Prism [Version 10.0.2 (232), GraphPad Software Inc., San Diego, USA] using a one-way ANOVA followed by a Tukes multiple comparison test.

### Thickness Measurements

The thickness of hydrated 8 mm BC and BC-AuNP composites was measured with a TIRF microscope (Ti-E, Nikon, Tokyo, Japan) at 10× magnification. Statistical analysis was performed with GraphPad Prism (Version 10.0.2 (232), GraphPad Software Inc., San Diego, USA), *n* = 6. Significance was tested with an unpaired *t*-test with Welch́s correction.

### Shape Recovery

Hydrated 8 mm BC and BC-AuNP composites were compressed to 15% or 50% strain using a Discovery HR-2 rheometer (TA Instruments, New Castle, DE, USA) equipped with an 8 mm parallel plate geometry at a compression speed of 10 μm/s at 25 °C (*n* = 3). The compression was maintained for 5 min until full relaxation was achieved; then, the samples were incubated for 24 h in MQ water at room temperature to allow for shape recovery. Sample thickness was measured prior to each compression using a TIRF microscope (Ti-E, Nikon, Tokyo, Japan), 10× magnification for a total of 5 days.

### Stress Relaxation

Hydrated 8 mm BC and BC-AuNP composites were sequentially compressed to 0.1, 0.5, 1, and 2 N axial force at 10 μm/s using a Discovery HR-2 rheometer (TA Instruments, New Castle, DE, USA) equipped with an 8 mm parallel plate geometry at 25 °C (*n* = 3). The samples were allowed to relax for 10 min at constant gap height to ensure full relaxation, while small amplitude oscillatory deformations were performed at 1 Hz and 0.01% strain. Compressive stress was recorded during relaxation steps and normalized against the stress at the end of the corresponding compression. Data were plotted with MATLAB R2022a (The MathWorks Inc., Natick, Massachusetts, United States), and the standard deviation was indicated as a shaded area in Figure S7. Storage and loss moduli were displayed as average and standard deviation at 5–10 min into the relaxation.

## Results and Discussion

**Figure 2:**
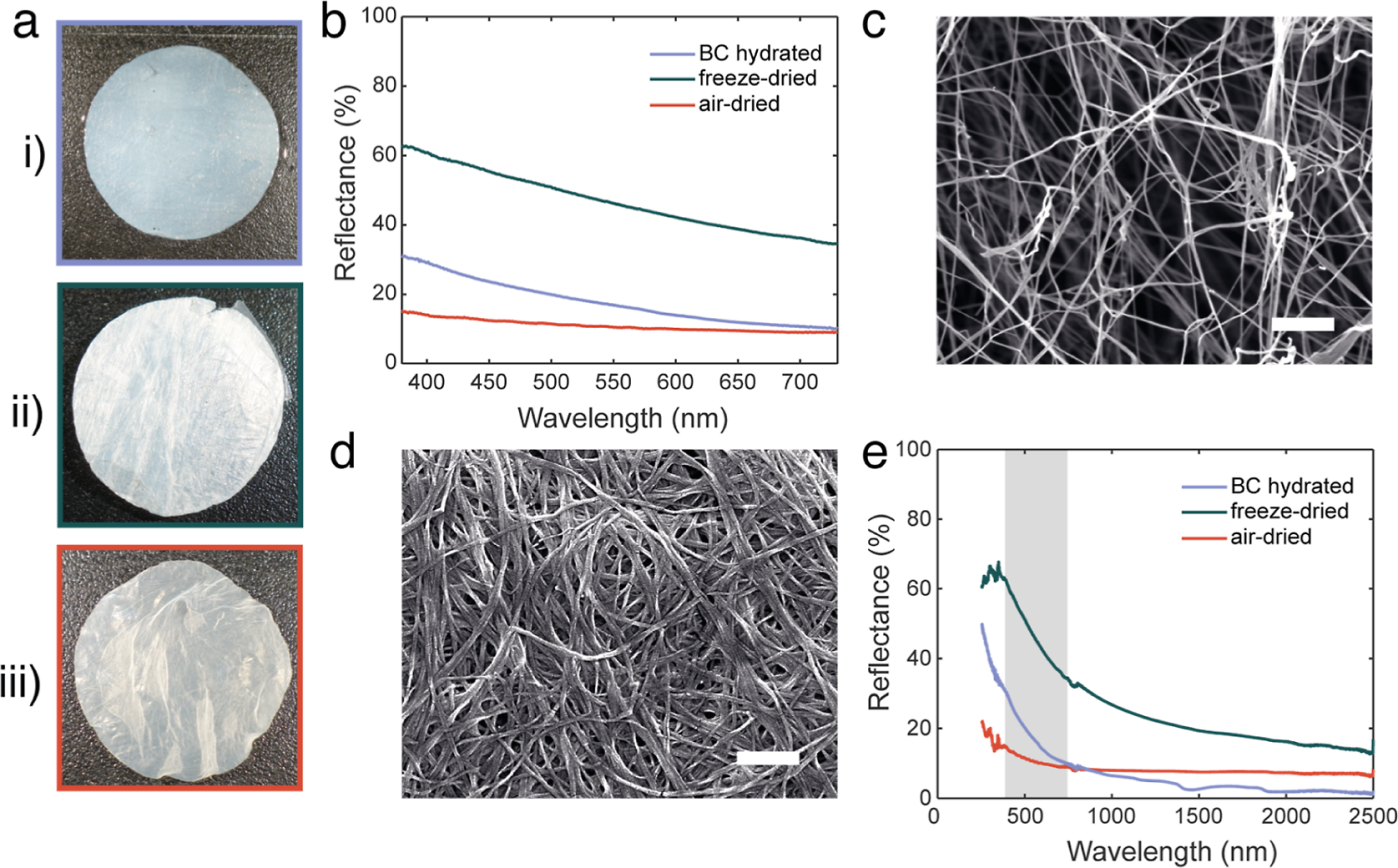
(a) Photographs of hydrated BC (i), freeze–dried BC (ii), and air-dried BC (iii). The diameter of the membranes is 2 cm. (b) Reflectance (DHR) spectra of hydrated BC, freeze-dried BC, and air-dried BC in the visible wavelength range. (c) SEM micrograph of freeze-dried BC, scale bar: 500 nm. (d) SEM micrograph of air-dried BC, scale bar: 500 nm. (e) Reflectance (DHR) spectra of hydrated BC, freeze–dried BC, and air-dried BC in the near-infrared wavelength range (visible wavelength range indicated with gray).

Native hydrated BC membranes are transparent (Figure 2a,i), showing a high degree of scattering in the visible spectrum. Due to the structure of the membranes, a large portion of the measured total DHR (Figure 2b) can be assumed to be diffusely reflected. The observed reflectance is primarily a result of the refractive index (*n*) contrast between the nanocellulose fibrils (*n* ≈ 1.49 at λ = 700 nm)^34^ and the surrounding medium and/or the medium filling the nanoporous structure of the materials, which for water is *n* ≈ 1.33 in the visible wavelength range.^35^ Freeze-drying keeps the fibrillar and porous 3D structure mostly intact and to some extent conserves the separation between the BC fibrils (Figure 2c). However, exchanging water for air (*n* ≈ 1.0) makes the refractive index contrast larger, resulting in increased light scattering (Figure 2b) and a whiter appearance (Figure 2a,ii). Air-drying of BC is a relatively slow process under ambient conditions and results in a collapse of the nanoporous structure (Figure 2d). The compact air-dried BC exhibits low reflectance both in the visible spectra and in the near-infrared (NIR) wavelength range (Figure 2e). The lower reflectance of air-dried BC is probably due to the close contact between the BC fibrils, resulting in a more homogeneous material compared to hydrated or freeze–dried BC, which has water or air-filled pores. However, small air pockets or irregularities in the air-dried structure are visible in scanning electron micrographs (Figure 2d) also for air-dried BC, resulting in a certain degree of scattering in the visible spectral range.

To increase the light-absorbing properties of BC, AuNPs (ø 12 ± 1 nm, Figure S1a) were adsorbed in the BC matrix. This was accomplished by submerging hydrated BC membranes in a highly concentrated AuNP suspension. The ionic strength of the suspensions was adjusted by using 10 mM citrate buffer (pH 6) to promote close contact between the AuNPs and the nanocellulose fibrils while maintaining colloidal stability of the NPs, resulting in efficient binding of the AuNPs to the BC nanofibrils. We have previously demonstrated that the adsorption process is dominated by short-range van der Waals interactions.^26^ Due to the porous structure and high-surface area of the water swollen BC, very large quantities of AuNPs could be adsorbed.

**Figure 3:**
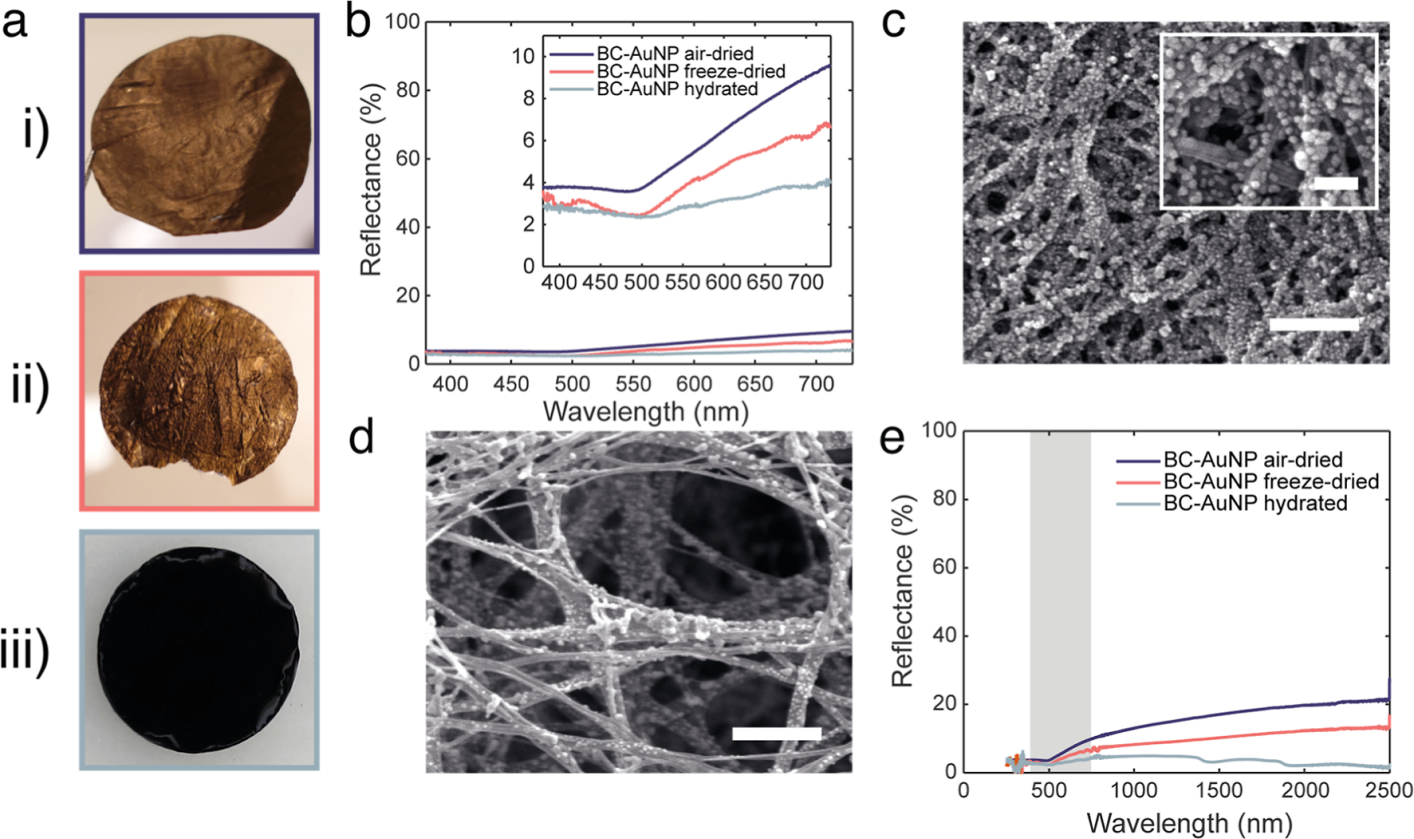
(a) Photographs of (i) air-dried, (ii) freeze–dried, and (iii) hydrated BC-AuNP. The diameter of the membranes is 2 cm. (b) Reflectance spectra of BC-AuNP in the visible wavelength range. (c) SEM micrograph of air-dried BC-AuNP, scale bar: 500 nm. Inset scale bar: 100 nm. (d) SEM micrograph of freeze-dried BC-AuNP, scale bar: 500 nm. (e) Reflectance spectra of BC-AuNP between 250–2500 nm (visible wavelength range indicated in gray).

During the initial adsorption phase, the BC membrane appeared bright red with a similar color to the suspended AuNPs, and the UV–vis spectra of the modified BC membranes demonstrated a sharp localized surface plasmon resonance (LSPR) band (Figure S1b, Supporting Information). By continually adding more AuNPs to the suspension, we observed that the NPs continued to adsorb in the BC membranes eventually resulting in a 120% increase in dry weight from 0.6 ± 0.1 mg of pristine BC to 1.3 ± 0.1 mg after AuNP adsorption (ø 8 mm membrane). The AuNPs remained firmly bound the BC membranes, and careful rinsing of the obtained BC-AuNP composites in water resulted in only minor dissociation of NPs. The stability of the BC-AuNP composites was confirmed by vigorously shaking the membranes in MQ overnight, after which the amount of released particles was measured to be approximately 0.1 ± 0.04% (Figure S1c, Supporting Information). No AuNP aggregation was seen during the assembly process, neither in the suspension nor in the BC membranes. However, due to the very high concentration of AuNPs in the BC membranes, the interparticle distance between individual AuNPs decreased significantly, resulting in near-field interactions. Consequently, we observed a major broadening of the LSPR band, eventually resulting in close to zero transmittance (total DHT) over the entire visible spectrum (Figure S2a, Supporting Information) and very low reflectance (Figure 3). An increase in transmittance was seen at wavelengths above 650 nm and reached about 40% at λ = 1250 nm (Figure S2b, Supporting Information).

Since BC is a water swollen hydrogel, the optical interactions between AuNPs adsorbed to different fibrils are likely limited. Drying makes the materials more compact, resulting in a smaller separation between individual fibrils. To explore the effect of the BC structure on the optical properties of the BC-AuNP composites, we subjected the hydrated BC-AuNP to either freeze–drying or air-drying. Interestingly, air-dried and freeze-dried BC-AuNP acquired a surface with a slight metallic sheen after drying (Figure 3a,i,ii), while hydrated BC-AuNP was profoundly black (Figure 3a,iii). This was seen also in the reflectance spectra for the three materials (Figure 3b) where the two dried materials acquired similarities to metallic gold reflections,^36^ likely due to the high content of AuNPs in the BC combined with a decrease in AuNP separation upon drying. The air-dried BC-AuNP, which had the least porous surface structure (Figure 3c), displayed the highest reflectance. Not surprisingly, freeze–dried BC-AuNP membranes had a similar structure to freeze–dried native BC (Figure 3d), and hence a more diffuse boundary to the surrounding medium and lower refractive index compared to the air-dried composites, contributing to the lower reflectance. The low reflectance of hydrated BC-AuNP membranes can be explained by the refractive index of water being between *n* for BC and air, thus reducing surface reflections. However, it is likely that the low reflectance of hydrated BC-AuNP is primarily due to the intact 3D structure. Freeze–drying, on the other hand, will to some extent disturb the 3D structure of the nanocomposites resulting in a smaller distance between fibrils, and subsequently the AuNPs, leading to a more pronounced bulk-like metal appearance.

**Figure 4:**
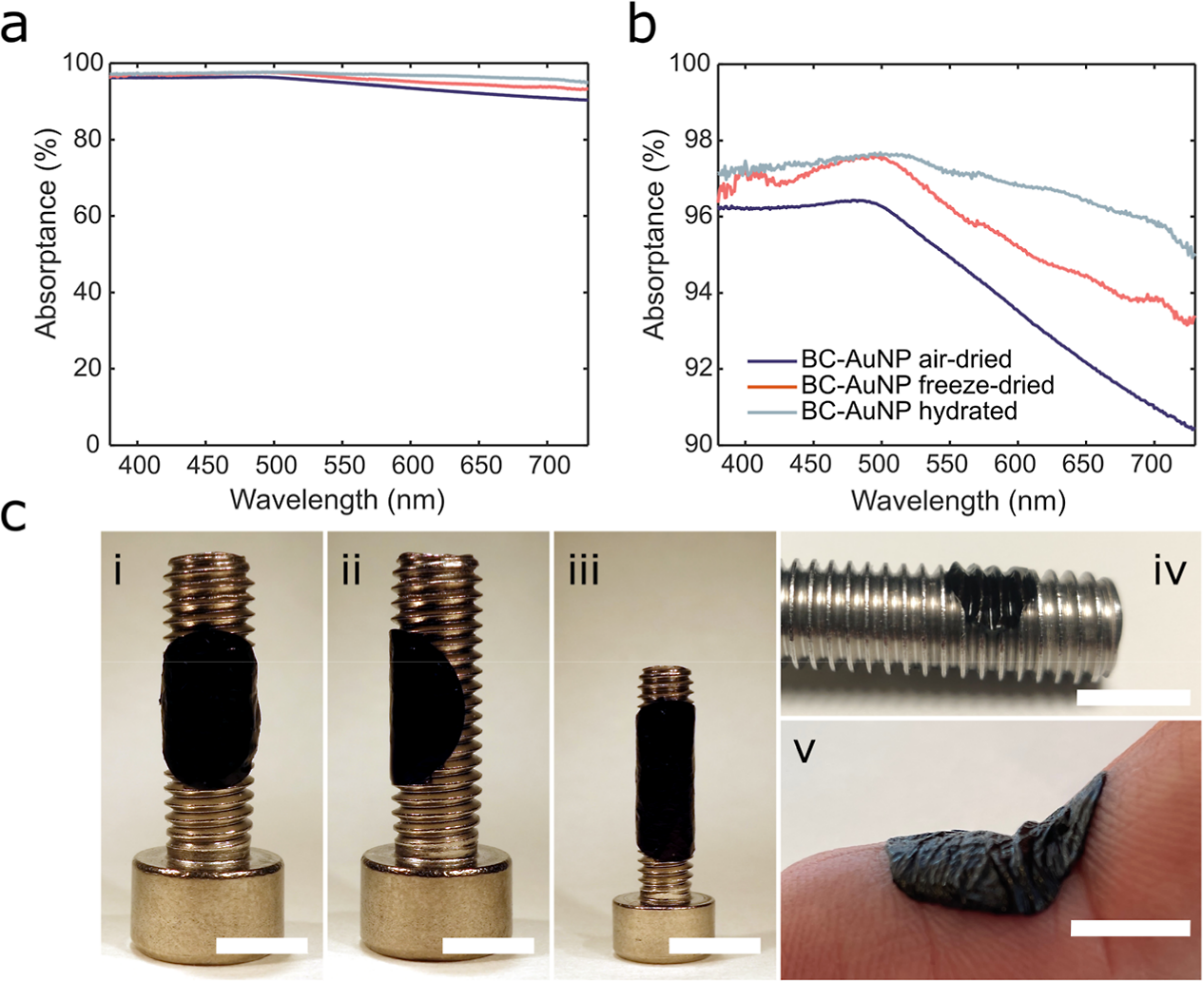
(a,b) Absorptance spectra of air-dried, freeze–dried, and hydrated BC-AuNP. (c) Photographs of hydrated BC-AuNP on a curved surfaces showing the conformability of the BC-AuNP composites on screws with a diameter of (i,ii) 5 mm, (iii) 3 mm, and (iv) 8 mm as substrates, and on a finger (v). The pitch of the threads in (iv) is 1.25 mm. Scale bars: (i–iii) 5 mm, (iv) 8 mm, and (v) 1 cm.

The transmittance of the air-dried BC-AuNP was virtually zero for the entire wavelength range between 250 and 2500 nm. The transmittance of freeze–dried BC-AuNP increased for wavelengths above 1250 nm, reaching a maximum transmittance of approximately 28% at λ = 2500 nm (Figure S3, Supporting Information). The increase in transmittance of freeze–dried BC-AuNP did not seem to have any influence on the reflectance of the material as there was very little difference in reflectance above λ ≈ 1250 nm compared to in the wavelength range between 250 and 1250 nm. Instead, reflectance slowly increased with increasing wavelength, similar to that in air-dried BC-AuNP. Thus, surface irregularities in freeze–dried BC-AuNP may result in areas with high reflectance and very low transmittance and vice versa. In contrast, air-dried BC-AuNP seems to have a more homogeneous surface, dominated by structures with very low transmittance and a certain amount of reflectance. Broadband absorbing materials show low reflectance and high absorptance. By measuring the reflectance and transmittance, the absorptance of BC-AuNP was calculated as *A* = (1 – *R* – *T*). Maximum absorptance was seen for hydrated BC-AuNP reaching about 97.7% at around λ ≈ 500 nm (Figure 4a,b). Air-dried and freeze–dried BC-AuNPs also showed a maximum in absorptance in this wavelength range with approximately 97.6% at λ = 491 nm and 96.4% at λ = 486 nm, respectively. For wavelengths between 750 and 1800 nm, freeze–dried and air-dried BC-AuNP absorbs light more extensively than hydrated BC-AuNP; however, there is a large influence of water at this wavelength region with absorptance peaks at λ ≈ 1450 and λ ≈ 1930 nm (Figure S4, Supporting Information).

**Figure 5:**
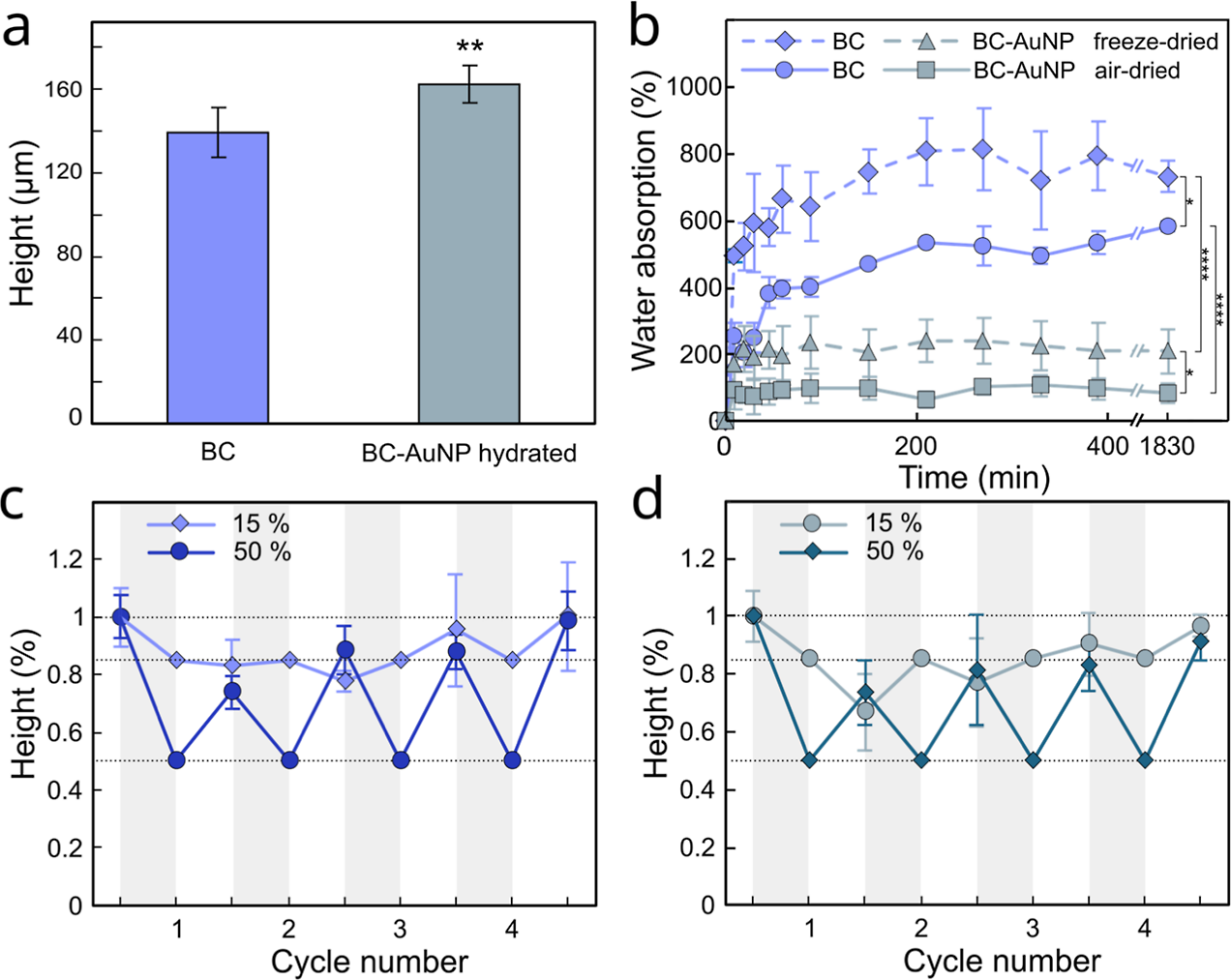
(a) Thickness of hydrated BC and BC-AuNP composites prior to drying. Significance was tested using unpaired *t*-test with Welch’s correction, *P* < 0.05, *n* = 6. (b) Water absorption of BC and BC-AuNP composites after freeze–drying and air-drying, *n* = 3. Statistical analysis was performed with an ordinary one-way ANOVA test complemented with Tukey’s multiple comparison test (*P < 0.1 and ****P < 0.0001). (c) Cycles of compression and rewetting of BC and (d) BC AuNP composites at two compression levels, 15% and 50%, and subsequent rehydration, normalized at hydrated pre-compression height, *n* = 3. Error bars show standard deviations.

Hydrated BC and BC-AuNPs showed a remarkable ability to conform to structured, curved, and uneven surfaces (Figure 4c). The mechanical flexibility of hydrated BC was not obviously affected by the adsorption of AuNPs although the incorporation of AuNPs resulted in a slight increase in thickness of the BC of about 16% from 139.3 ± 11.7 μm of pristine BC to 162.5 ± 9.2 μm for BC-AuNP composites (Figure 5a). The increase in thickness is likely caused by the increase in osmotic pressure from ions and counterions associated with the AuNPs adsorbed to the BC fibrils, resulting in swelling. Drying made the materials more brittle; however, they still retained substantial flexibility, and the membranes could be wrapped around curved surfaces (Figure S5, Supporting Information). Once dried, rehydration resulted in swelling and regain of the initial flexibility, although the drying had a noticeable impact on the water absorption capacity of both materials. Air-drying resulted in significantly more compact materials and a lower degree of reswelling upon hydration, corresponding to 85.7% for BC-AuNP compared to 583.3% for native BC at equilibrium. Freeze–drying resulted in a less compact structure and thus higher water absorption capacity and a higher degree of reswelling, corresponding to 209.8% for BC-AuNP and 732.2% for native BC at equilibrium, Figure 5b. The presence of AuNPs in the nanocellulose network clearly hindered water absorption and reswelling of the materials, likely due to a combination of a decrease in the membrane pore size and the formation of additional stabilizing interfibrillar interactions mediated by the AuNPs, leading to a reinforcement of the material that hindered reswelling and also made the membranes more mechanically robust.

Due to the high water content of the hydrated materials, a slight pressure had to be applied for the materials to conform to and retain their shape on surfaces with sharp and uneven features, such as the narrow grows of, for example, a screw (Figure 4c,iv). No obvious changes in the optical properties were seen when pressure was applied, and the materials retained their pronounced black appearance. To further characterize the mechanical properties and shape recovery of the hydrated BC and BC-AuNP composites when subjected to mechanical load, we investigated their response to moderate axial deformations. The materials were iteratively compressed to 85% or 50% of their initial height, and their shape recovery was evaluated over four compression cycles. Low deformation levels (15% compression) resulted in relatively slow but almost complete shape recovery (100 ± 19% for BC and 97 ± 4% for BC-AuNP) over the course of the experiment (Figure 5c,d). Interestingly, higher deformation levels (50% compression) resulted in a faster recovery reaching 74–99% and 73–91% of the original thickness, for BC and BC-AuNP, respectively. The faster recovery for higher compression levels is likely the result of internal charge repulsion in the materials and the increase in osmotic pressure due to the expulsion of water, promoting rapid water influx and shape recovery after relaxation of the compression. These data clearly indicate that the materials could withstand compression levels ≤50% without permanent deformation. Under these conditions, the network topology was apparently retained, and a limited number of new interfibrillar connections were formed.

**Figure 6:**
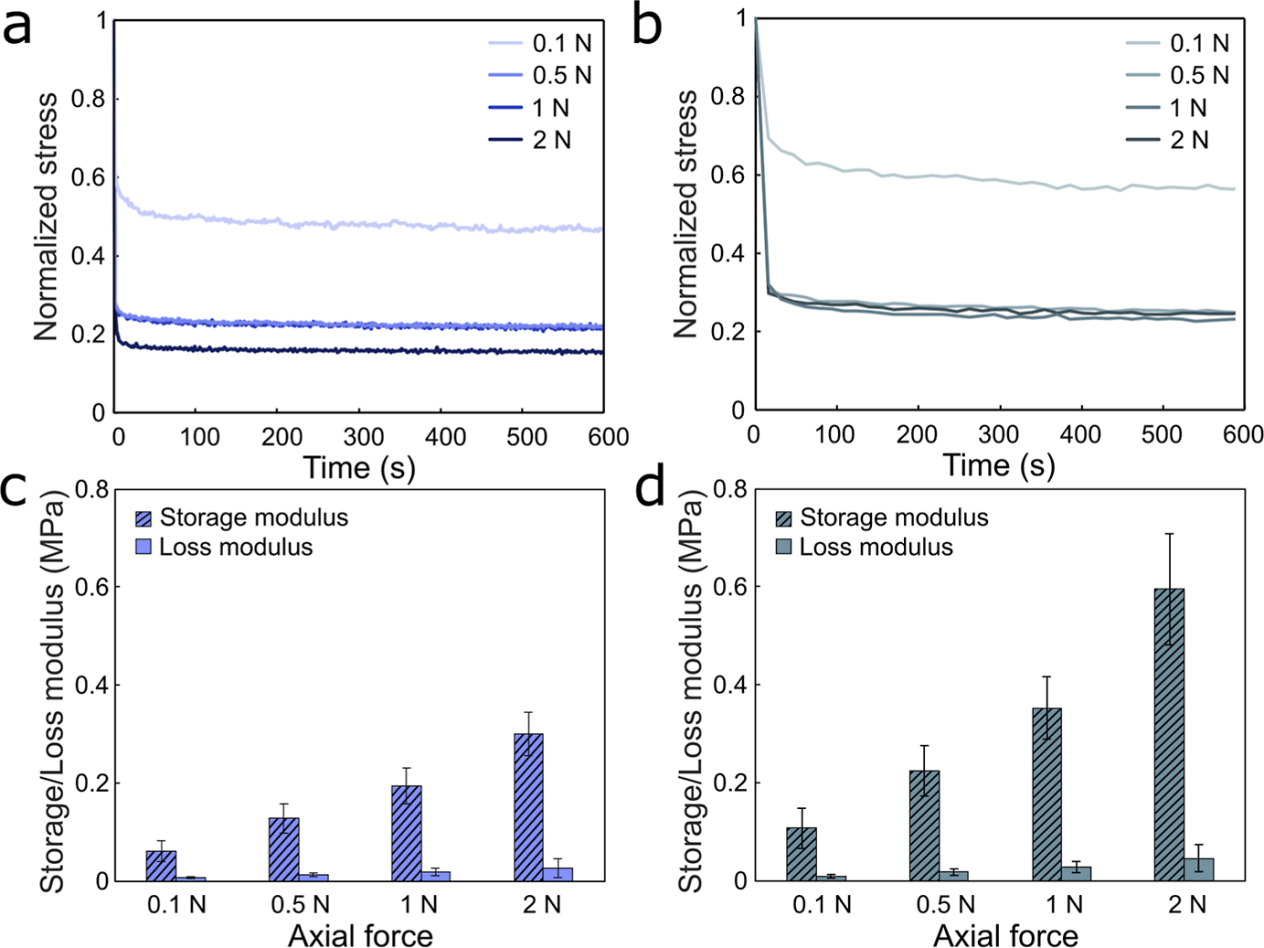
Normalized stress relaxation curves of hydrated (a) BC and (b) BC-AuNP composites subjected to 0.1–2 N axial compression. Average values are displayed (*n* = 3). Storage (*G*′) and loss modulus (*G*″) of hydrated (c) BC and (d) BC-AuNPs composites recorded during the last 5 min of the relaxation step. Data displayed as mean and standard deviation, *n* = 3.

To explore whether the reinforcement of the materials that was observed after drying also could be induced by mechanical compression, we next investigated the effect of higher deformation levels on hydrated BC and BC-AuNPs by performing compressive rheology experiments. Compression forces ranging from 0.1 to 2 N, equivalent to a compressive strain of 45 ± 8–85 ± 4% and 39 ± 5–76 ± 5% for BC and BC-AuNP composites, respectively, were applied to the samples. During compression, the water content of the hydrogels was reduced as water freely exited the materials in the radial direction. Following compression, a 10 min relaxation step was introduced, during which axial stress was recorded. The relaxation speed was nearly identical for BC (Figure 6a) and BC-AuNP composites (Figure 6b). None of the materials displayed full relaxation, and after 10 min, pristine BC displayed 53%, 79%, 78%, and 84% recovery after being subjected to a compression force of 0.1, 0.5, 1, and 2 N, respectively (Figures 6a and S7a). BC-AuNP displayed slightly lower relaxation for the same compression levels corresponding to 51%, 80%, 81%, and 81%, respectively (Figures 6b and S7b). These findings show that BC possessed a load-bearing capacity due to its ability to store elastic energy that was further enhanced by the incorporation of AuNPs.

The material stiffening resulting from compression was evaluated by recording the storage and loss moduli at different compression levels (Figure 6c,d). The increase in the storage modulus with increasing compression confirms the strengthening of the network. This was especially pronounced for the BC-AuNP samples, demonstrating that the adsorbed AuNPs could form new interactions between adjacent fibrils during compression, as discussed above. Due to this reinforcement effect, samples subjected to compression strains >50% gradually lost their capacity to recover their shape. However, no apparent changes in the broadband absorptance were observed, allowing for the materials to be draped and forced to conform to irregular surface structures with retained optical properties.

## Conclusions

In summary, we demonstrate a simple strategy for generating soft and mechanically flexible broadband absorbing materials by self-assembly of AuNPs in BC. The materials showed an absorptance > 96% with differences in reflectance depending on the state of hydration. Hydrated BC-AuNPs showed the highest absorptance in the visible wavelength range and lowest reflectance compared to air- and freeze–dried BC-AuNP. Air-drying resulted in the optically most homogeneous materials but highest reflectance, whereas hydrated BC-AuNP showed very low reflectance of light in both the visible and the near-infrared wavelength range. All materials retained high mechanical flexibility and could conform to a curved surface and highly irregular surface structures, including skin. The materials could withstand compression levels ≤50% without permanent deformation. Higher strains resulted in the formation of new interfibrillar connections mediated by the adsorbed AuNPs and a reinforcement of the material that prevented shape recovery. Thus, applying a certain amount of pressure enabled the materials to adopt and keep the shape of sharp irregular surface structures. The possibility of using self-assembly to adsorb large quantities of colloidal AuNPs in a sustainable nanocellulose scaffold represents a novel and cost-effective strategy for generating broad band light-absorbing materials with unique mechanical properties.

## Supporting Information Available

UV–vis spectra, transmittance spectra and absorptance spectra of BC-AuNP, additional photographs of BC and BC-AuNP, description of setup for transmittance and reflectance measurement, and stress relaxation curves (PDF)
