# Supplementary material for: Self-Assembly of Soft and Conformable Broadband Absorbing Nanocellulose-Gold Nanoparticle Composites
Source: ACS Appl Mater Interfaces. 2024 Sep 23;16(39):52894–901. doi: 10.1021/acsami.4c10244 (PMC11450681; doi:10.1021/acsami.4c10244)
Supplement: Supplementary file 1 — am4c10244_si_001.pdf [file am4c10244_si_001.pdf]

Supporting Information

Self-Assembly of Soft and Conformable Broadband Absorbing Nanocellulose-Gold Nanoparticle Composites

Olof Eskilson[1], Elisa Zattarin[1], Jennifer Silander[2], Tomas Hallberg[2], Christina Åkerlind[2], Robert Selegård[1], Kenneth Järrendahl[3], Daniel Aili[1,]*

[1] Laboratory of Molecular Materials, Division of Biophysics and Bioengineering, Department of Physics, Chemistry, and Biology, Linköping University, 58183 Linköping, Sweden.

[2] FOI – Swedish Defence Research Agency, Department of Electromagnetic Signatures, 583 30 Linköping, Sweden.

[3]Thin Film Physics Division, Department of Physics, Chemistry, and Biology, Linköping University, 58183 Linköping, Sweden.

*Corresponding author: daniel.aili@liu.se

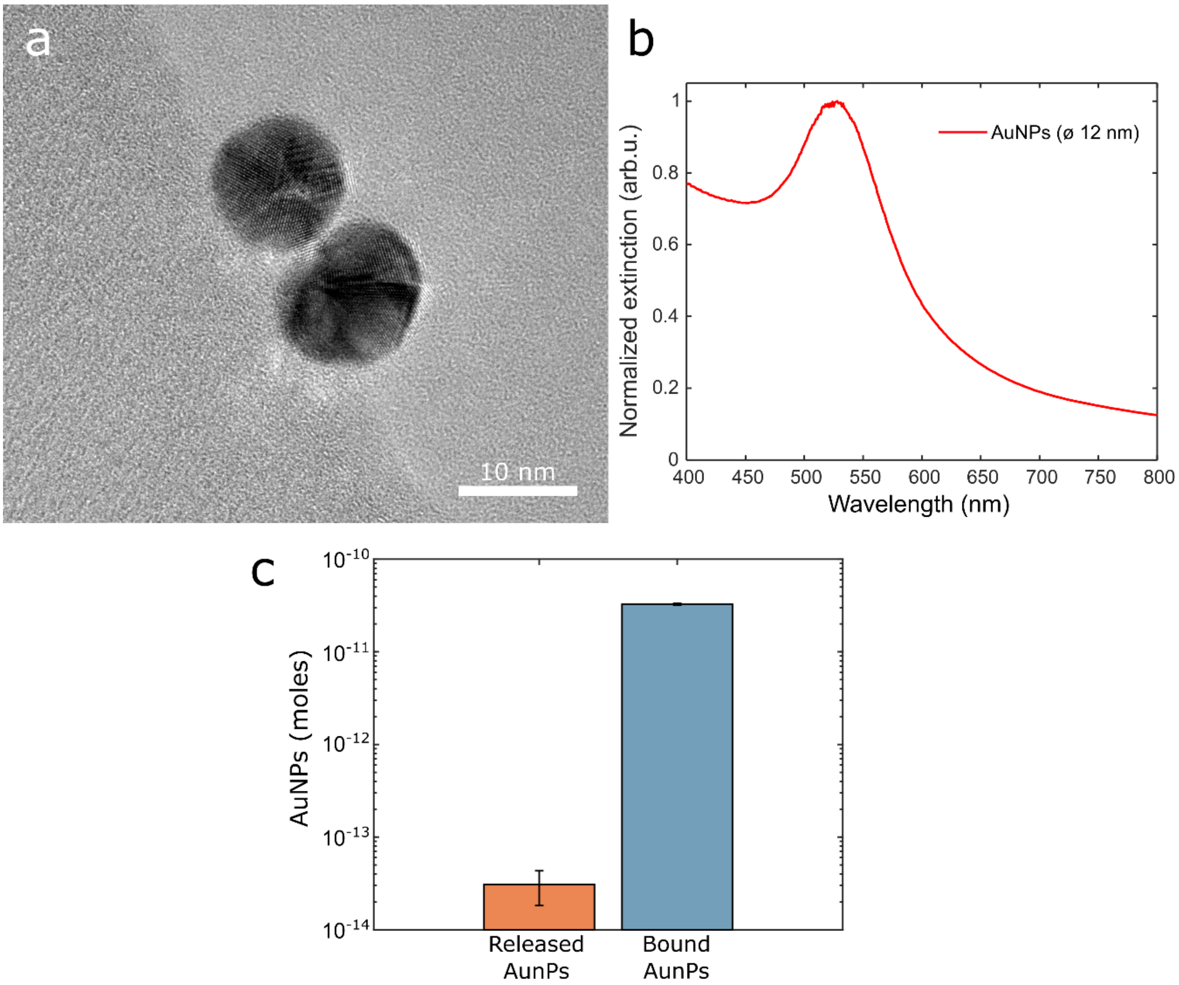

Figure S1. a) Transmission electron micrograph of AuNPs (ø 12 ± 1 nm). **b)** UV-vis spectrum of a ø 6 mm BC-AuNP membrane obtained by incubating BC in 1 mL 13 nM AuNP suspension for 5 days. **c)** Released and bound AuNPs after stability test (n=5), error bars show standard deviation.

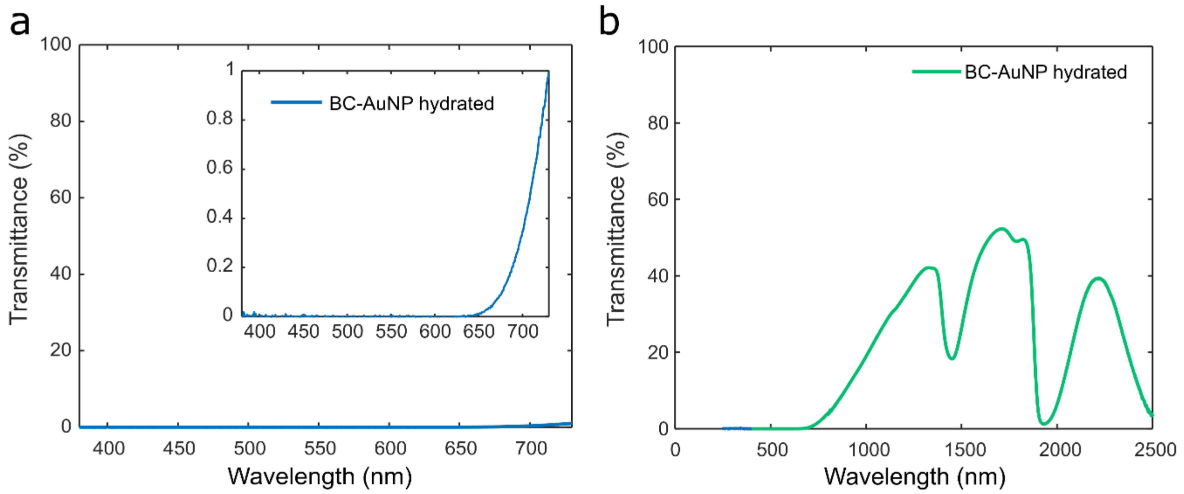

Figure S2. Transmittance (DHT) spectra of hydrated BC-AuNP in the **a)** visible range, and in the **b)** extended range (250–2500 nm).

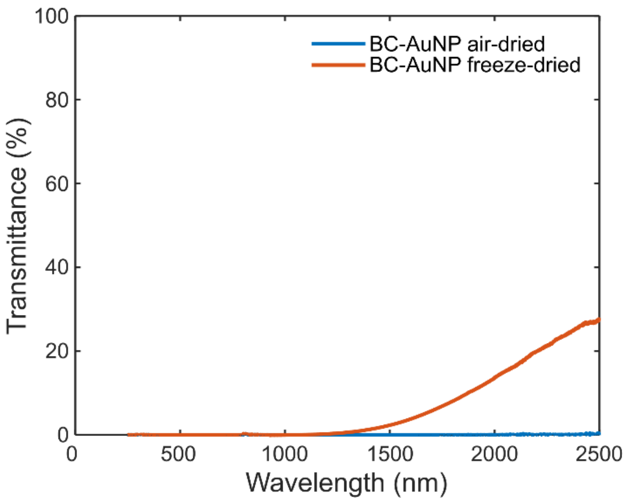

Figure S3. Transmittance spectra (DHT) of air-dried and freeze-dried BC-AuNP.

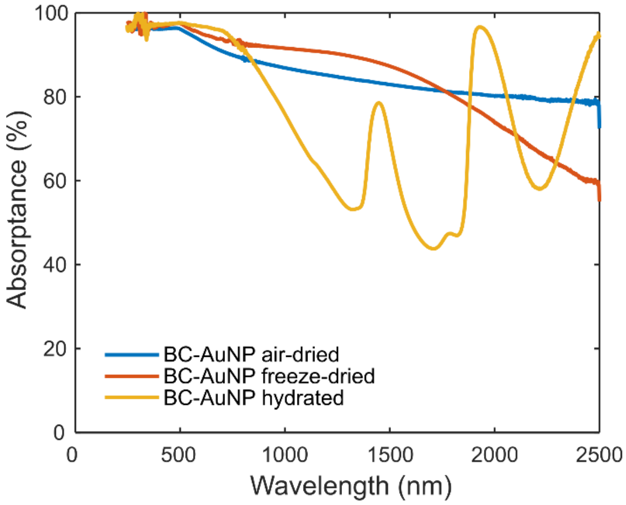

Figure S4. Absorptance spectra of BC-AuNP.

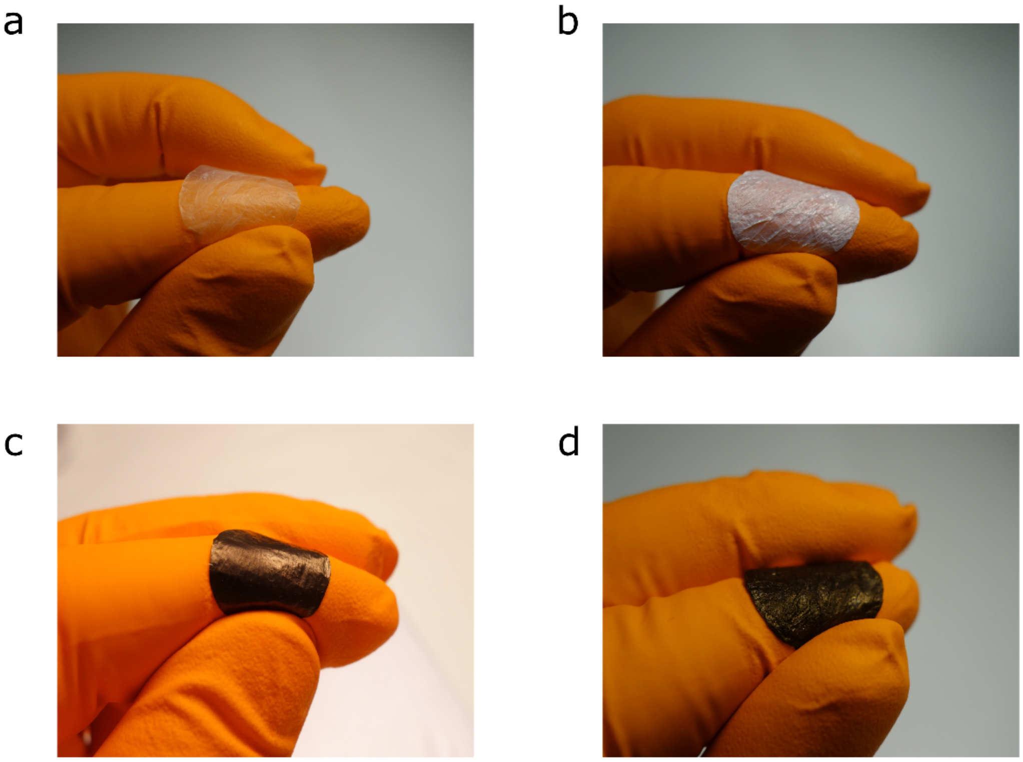

Figure S5. Photos of **a)** air-dried BC, **b)** freeze-dried BC, **c)** air-dried BC-AuNP, and **d)** freeze-dried BC-AuNP.

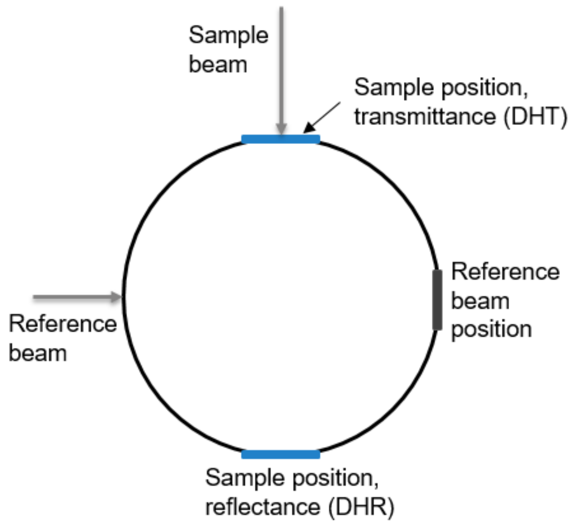

Figure S6. Schematic illustration of the setup for transmittance and reflectance measurement.

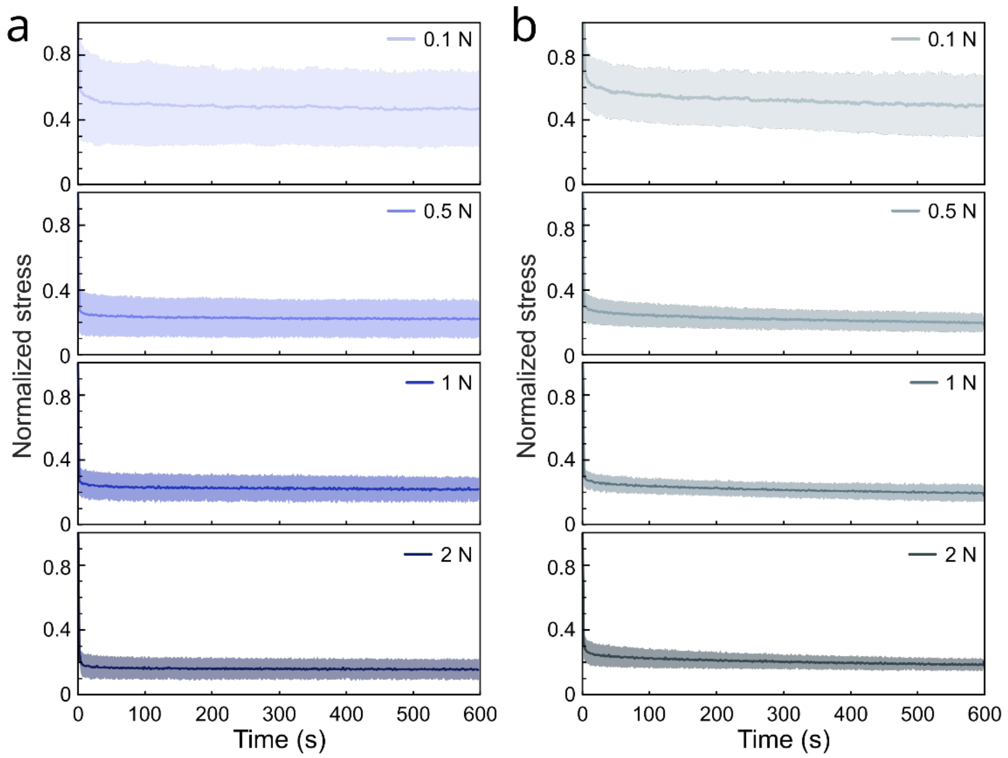

Figure S7. Stress relaxation curves of **a)** BC and **b)** BC-AuNP cat 0.1 N, 0.5 N, 1 N and 2 N compressive force. Curves are displayed as mean ± standard deviation, n = 3.
